# Associations between tamoxifen, estrogens, and FSH serum levels during steady state tamoxifen treatment of postmenopausal women with breast cancer

**DOI:** 10.1186/1471-2407-10-313

**Published:** 2010-06-21

**Authors:** Jennifer Gjerde, Jürgen Geisler, Steinar Lundgren, Dagfinn Ekse, Jan Erik Varhaug, Gunnar Mellgren, Vidar M Steen, Ernst A Lien

**Affiliations:** 1 Hormone Laboratory, Haukeland University Hospital, Bergen, N-5021, Norway; 2 Section for Endocrinology, Institute of Medicine, University of Bergen, Bergen, N-5021, Norway; 3 Department of Oncology, Haukeland University Hospital, Bergen, N-5021, Norway; 4 Section for Oncology, Institute of Medicine, University of Bergen, Bergen, N-5021, Norway; Faculty Division Akerhus University Hospital, University of Oslo, Oslo, N-0316, Norway; 5 Section of Oncology, Department of Medicine, Akershus University Hospital, Lørenskog, N-1478, Norway; 6 Department of Oncology, St. Olavs University Hospital, Trondheim, N-7006, Norway; 7 Department of Cancer Research and Molecular Medicine, Faculty of Medicine, Norwegian University of Science and Technology, Trondheim, N-7006, Norway; 8 Department of Surgery, Haukeland University Hospital, Bergen, N-5021, Norway; 9 Department of Surgical Sciences, University of Bergen, Bergen, N-5021, Norway; 10 Dr. E. Martens Research Group for Biological Psychiatry, Department of Clinical Medicine, University of Bergen, Bergen, N-5021, Norway; 11 Center for Medical Genetics and Molecular Medicine, Haukeland University Hospital, Bergen, N-5021, Norway

## Abstract

**Background:**

The cytochrome P450 (CYP) enzymes 2C19, 2D6, and 3A5 are responsible for converting the selective estrogen receptor modulator (SERM), tamoxifen to its active metabolites 4-hydroxy-tamoxifen (4OHtam) and 4-hydroxy-*N*-demethyltamoxifen (4OHNDtam, endoxifen). Inter-individual variations of the activity of these enzymes due to polymorphisms may be predictors of outcome of breast cancer patients during tamoxifen treatment. Since tamoxifen and estrogens are both partly metabolized by these enzymes we hypothesize that a correlation between serum tamoxifen and estrogen levels exists, which in turn may interact with tamoxifen on treatment outcome. Here we examined relationships between the serum levels of tamoxifen, estrogens, follicle-stimulating hormone (FSH), and also determined the genotypes of CYP2C19, 2D6, 3A5, and SULT1A1 in 90 postmenopausal breast cancer patients.

**Methods:**

Tamoxifen and its metabolites were measured by liquid chromatography-tandem mass spectrometry. Estrogen and FSH levels were determined using a sensitive radio- and chemiluminescent immunoassay, respectively.

**Results:**

We observed significant correlations between the serum concentrations of tamoxifen, *N*-dedimethyltamoxifen, and tamoxifen-*N*-oxide and estrogens (p < 0.05). The genotype predicted CYP2C19 activity influenced the levels of both tamoxifen metabolites and E1.

**Conclusions:**

We have shown an association between tamoxifen and its metabolites and estrogen serum levels. An impact of CYP2C19 predicted activity on tamoxifen, as well as estrogen kinetics may partly explain the observed association between tamoxifen and its metabolites and estrogen serum levels. Since the role of estrogen levels during tamoxifen therapy is still a matter of debate further prospective studies to examine the effect of tamoxifen and estrogen kinetics on treatment outcome are warranted.

## Background

Estrogens play a key role in breast cancer development. The selective estrogen receptor modulator (SERM) tamoxifen has been used in breast cancer treatment and prevention. It may act as a full estrogen agonist, partial agonist or antagonist depending on the dose, species, sex or target organ [1]. Tamoxifen is regarded as a pro-drug since two of its metabolites, 4-hydroxytamoxifen (4OHtam) and 4-hydroxy-*N*-demethyltamoxifen (4OHNDtam, endoxifen), both have estrogen receptor affinity markedly exceeding that of tamoxifen itself [2,3]. The 4OHNDtam is considered the main active metabolite of tamoxifen, since it has 100-fold higher affinity for the estrogen receptor (ER) than tamoxifen and is 10-fold higher in serum levels than 4OHtam [4-7]. These potent metabolites are converted from tamoxifen through the cytochrome P450 (CYP) enzymes 2C19, 2D6, and 3A5. They are conjugated and deactivated through sulfotransferase (SULT) 1A1 [8,9] and UDP-glucuronyltransferases. The inter-individual variations of the activity of these enzymes due to genetic polymorphisms could therefore be predictors of outcome during tamoxifen treatment considering their influence on the concentration of the active metabolites 4OHNDtam and 4OHtam [7]. However, the results from clinical studies are partly contradictory [10-20]. The conflicting results may be explained by differences in study designs, including size, different genetic models for the assessment of phenotypes, or dosing regimens.

Tumors that initially respond to tamoxifen treatment develop resistance over time [21]. Several mechanisms resulting in tamoxifen resistance have been suggested. Among others the findings of Berstein *et al *that long-term exposure to tamoxifen induces hypersensitivity to 17β-estradiol (E2) suggests that E2 levels may be of importance when resistance developes [22].

Hormonal changes involving the elevation of serum concentrations of follicle-stimulating hormone (FSH) and cessation of E2 levels during and after the menopause are related to the frequency of hot flashes [23,24], which in turn have been suggested as a predictor of tamoxifen efficacy [25,26]. Patients carrying functional CYP2D6 alleles been reported to have a higher incidence of hot flashes, higher levels of the active metabolites of tamoxifen, and better outcome during tamoxifen treatment [7,25,26].

**Figure 1:**
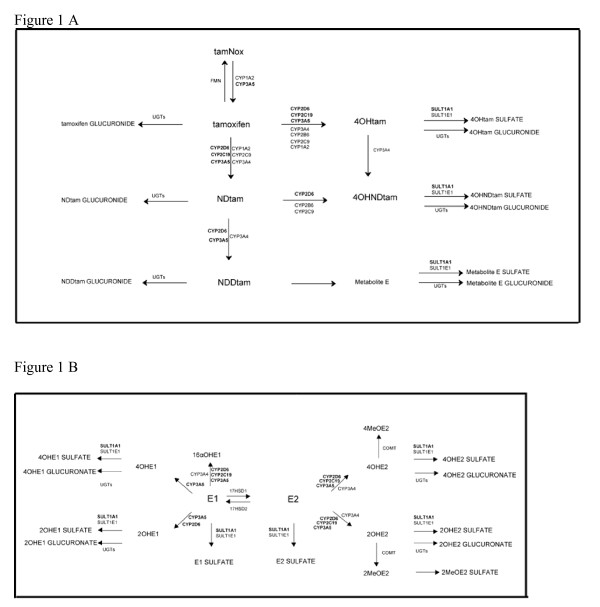
**Schematic representation of (A) tamoxifen and (B) estradiol metabolism and the enzymes involved**. 4OHtam, 4-hydroxytamoxifen; 4OHNDtam, 4-hydroxy-*N*-demethyltamoxifen; NDtam, *N*-dedimethyltamoxifen; NDDtam, *N*-dedimethyltamoxifen; tamNox, tamoxifen-*N *-oxide; CYP, cytochrome P450; SULT, sulfotransferase; UGT, Uridine 5'-diphospho-glucuronosyltransferase; E2, estradiol; E1, estrone; E1S, estrone sulphate; 4OHE2, 4-hydroxyE2; 2OHE2, 2-hydroxyE2; 4MeOE2, 4-methoxyE2; 2MeOE2, 2-methoxyE2; 4OHE1, 4-hydroxyE1; 2OHE1, 2-hydroxyE1; 4MeOE1, 4-methoxyE1; 2MeOE1, 2-methoxyE1; 16αOHE1, 16α-hydroxyE1; Catechol-*O*-methyl transferase, COMT. Bolded texts indicate tamoxifen and estrogen metabolizing enzymes investigated.

Tamoxifen and estrogens are both partly metabolized by the enzymes CYP2C19, 2D6, 3A5, and SULT1A1 (Figure 1) [27]. Therefore, we hypothesized that these genotypes that are proposed predictors for response to tamoxifen influence estrogen metabolism and that correlations between serum tamoxifen and estrogen levels exist. Here, we examined relationships between the serum levels of tamoxifen, estrogens, follicle-stimulating hormone (FSH), and sex hormone-binding globulin (SHBG) in postmenopausal breast cancer patients. We also determined the genotypes of CYP2C19, 2D6, 3A5, and SULT1A1. We observed an association between tamoxifen and its metabolites and estrogen serum levels. The CYP2C19 predicted activity influenced both tamoxifen and estrogen kinetics.

## Methods

### Patients

From a study on tamoxifen metabolism in pre- and postmenopausal women adjuvantly treated with tamoxifen 20 mg daily, we included exclusively postmenopausal women in order to examine patients with stable estrogen and FSH levels [7]. Postmenopausal status was defined as FSH levels > 20 IU/L and E2 levels < 70 pmol/L, using the automated analyzer Immulite 2000 (Diagnostic Products Corporation, Los Angeles, CA). All patients with estrogen receptor and/or progesterone receptor-positive tumors gave their written informed consent to participate in the investigation. The protocol was approved by the National Committee for Medical and Health Research Ethics (NEM). Due to the long half-life of tamoxifen and to ensure that these patients had reached steady-state drug levels, only patients treated for at least 80 days were included in the study. All patients were informed to avoid tamoxifen ingestion on the morning of blood sampling. Whole blood and serum samples were immediately frozen and kept at -80°C until analysis.

### Determination of tamoxifen and its metabolites concentrations

Tamoxifen citrate and 4OHtam were purchased from Sigma-Aldrich (Steinheim, Germany), the internal standard deuterated5-tamoxifen (D_5_tam) and tamoxifen-*N*-oxide (TamNox) from Beta Chem Inc. Life Science, Research & Development (Kansas City, US), 4OHNDtam from Sintef Materials and Chemistry (Oslo, Norway). *N*-demethyltamoxifen (NDtam) and *N*-dedimethyltamoxifen (NDDtam) were gifts from Imperial Chemical Industries, PLC Pharmaceutical divisions (Macclesfield, UK). A high-pressure liquid chromatography-tandem mass spectrometry system was used for the determination of tamoxifen, 4OHtam, 4OHNDtam, NDtam, NDDtam and tamNox in serum [7,28].

### Determination of hormones and SHBG concentrations

FSH and SHBG levels were measured using the Immulite 2000 (Diagnostic Products Corporation, Los Angeles, CA). Serum levels of E2, estrone (E1), and estrone sulfate (E1S) were determined using a novel and highly sensitive radioimmunoassay where the detection limit for E2, E1, and E1S were 0.67 pmol/L, 1.14 pmol/L and 0.55 pmol/L, respectively [29].

### CYP2C19 genotype determination

Genomic DNA was extracted and purified from the whole blood samples using the QIAmpR DNA Blood Mini Kit as described in the manufacturer's (QIAmpR DNA Blood Mini Kit) handbook (Qiagen, Hilden, Germany).

CYP2C19 *2 (rs 4244285) and *3 were detected using the LightMix assay kit (TiBMolBiol, Berlin, Germany). CYP2C19 *17 (806C_T and 3402C_T) were determined by DNA sequencing and hybridization probe-based real-time PCR assay (Roche). For CYP2C19*17 (806C_T), we used the primer 5'-AGTGGTTCTATTTAATGTGAAGCCT -3' (forward) and 5'-GGTCTCCCTTTCCCATTTG -3' (reverse). The PCR product was bidirectionally sequenced using Big Dye Terminator version 3.1 Cycle Sequencing kit according to the manufacturer's recommendations on an ABI Prism 3700 DNA Analyzer (Applied Biosystems). The CYP2C19*17 (3402C_T) variant allele was determined by a hybridization probe-based real-time PCR assay (Roche), using the primer 5'-ATATCTGATAAATGATGGCTATCC-3' (forward) and 5'-CTGCAAGCTAAAACCCC-3' (reverse). The detection probe used was 5'-LC Red640-GACCCGTTGCCCATTTTTTTAATCAA--PH and the anchor probe was 5'-TGTCTTCTTGGTGAGATGTCTGTTC--FL (TIB MolBiol, Berlin, Germany).

### CYPD6 genotype determination

Genomic leukocyte DNA was extracted from EDTA-anticoagulated whole blood using the QIAamp DNA Blood Mini Kit (Qiagen, Hilden, Germany). The CYP2D6 genotypes were tested previously and subdivided into four groups and ranked according to their predicted increasing enzymatic activity: poor metaboliser (PM) [subjects with any combination of two nonfunctional variant type (Vt) alleles *3, *4, *5 and *6], intermediate metabolizer (IM) wild type/variant type CYP2D6, wild type/wild (Wt/Wt) type CYP2D6, and ultra rapid metabolizer (UM) [heterozygous Vt allele *2 × 2] [7].

### CYP3A5 genotype determination

The most common CYP3A5 variant (*3) was tested to identify patients with low CYP3A5 protein expression. This allele is present in approximately 20% Asian, 50% African-American, and 90% Caucasian populations. The CYP3A5*3 variant allele was determined by a hybridization probe-based real-time PCR assay (Roche, Germany), using the primer 5'-TTTGCCTCTTTGTACTTCTTCATC-3' (forward) and 5'-TAGTTGTACGACACACAGCAACC-3' (reverse). The detection probe was 5'-LC Red640-GTTTGGACCACATTACCCTTCATC--PH and the anchor probe was 5'-CTTTTGTCTTTCAATATCTCTTCCC--FL (TIB MolBiol, Berlin, Germany).

### SULT1A1 genotype determination

We screened for the SULT1A1*2 variant allele with a hybridization probe-based real-time PCR assay. To verify these results, a PCR-based restriction fragment length polymorphism assay and bi-directional sequencing were used as previously published [7]. The SULT1A1 copy number was estimated by calculating the height and area ratio of the 210 bp amplicon of SULT1A1 to the reference 205 bp amplicon of SULT1A2 as described by Hebbring *et al *[30].

### Statistical analyses

CYP2C19 was sub-divided into five groups and ranked according to their predicted increasing enzymatic activity: *2/*2 were subjects homozygous for the variant allele CYP2C19*2, *1/*2, were subjects heterozygous for the variant allele CYP2C19*2, homozygous wild type allele CYP2C19*1, CYP2C19*2/*17, *1/*17 were subjects heterozygous for the CYP2C19*17 variant allele, and *17/*17 were subjects homozygous for the CYP2C19*17 variant allele.

CYP2D6 was sub-divided into four groups and ranked according to their predicted increasing enzymatic activity: PM, IM, Wt/Wt, and UM. CYP3A5 was divided into CYP3A5*3/*3 and CYP3A5*1/*3 groups. SULT1A1 genotype and gene dosage were also sub-divided into groups and ranked according to their predicted increasing enzymatic activity: *2/*2, *1/*2 and *1/*1 and increasing copy number, respectively. Statistical analyses were conducted using SPSS statistical software (version 12; SPSS, USA). All statistical tests were two-sided.

The serum levels of tamoxifen and its metabolites, estrogens, FSH, and SHBG are given as median values and inter-quartiles ranges. The Spearman rank correlation coefficient was calculated in order to assess the association between two variables. Bonferroni correction was applied to take account of multiple comparisons. All P values were two sided. A multivariate logistic regression model was applied. The serum levels of tamoxifen and its metabolites (tamoxifen, 4OHtam, 4OHNDtam, NDtam, NDDtam, and TamNox), metabolic ratios (4OHtam/tam, tamNox/tam, NDtam/tamNox, and 4OHNDtam/NDtam) and estrogens (E2, E1, and E1S) were dependent variables and all genotypes (CYP2C19, CYP2D6, CYP3A5, SULT1A1, and SULT1A1 copy number), FSH, and SHBG were explicative variables. All tests were conducted at the P = 0.05 level of significance.

## Results

### Patients

Ninety patients (median age 62 years) were enrolled in this study. The median duration of tamoxifen treatment of patients included was 571 days (Table 1). The median [range] serum concentrations of E2 and FSH were 9.4 [1.3-54.7] pmol/L and 38.2 [20.3-108.0] IU/L, respectively, were consistent with post-menopausal values (Table 1). The serum level of SHBG was 81 [27-173]. Notably, we detected tamoxifen and its metabolites in all serum samples [range 31-247 ng/ml for tamoxifen] indicating good compliance for the patients included (Table 1).

**Table 1:** Age, duration of treatment, and biochemical and pharmacological parameters (n = 90)

Variables	Median [Range]		
Age	62	[48-85]	Year
Tamoxifen duration	571	[84-4380]	Days
E2^1^	9.4	[1.3 - 54.7]	pmol/L
E1	98.6	[29.3 - 355.6]	pmol/L
E1S	582	[30-2923]	pmol/L
FSH	38.2	[20.3 - 108.0]	IU/L
SHBG	81	[27-173]	nmol/L
Tam	96	[31-247]	ng/ml
4OHtam	5.8	[2.7 - 17.2]	ng/ml
4OHNDtam	50.7	[25.1 - 184.8]	ng/ml
NDtam	230	[116-596]	ng/ml
NDDtam	39.8	[15.7 - 93.4]	ng/ml
tamNox	9.6	[3.5 - 37.9]	ng/ml

^1^E2, estradiol; E1, estrone; E1S, estrone sulfate; FSH, follicle stimulating hormone; SHBG, sex hormone binding globulin; tam, tamoxifen; 4OHtam, 4-hydroxytamoxifen; 4OHNDtam, 4-hydroxy-N-demethyltamoxifen; NDtam, N-demethyltamoxifen; NDDtam, N-dedimethyltamoxifen; tamNox, tamoxifen-N-oxide.

### Frequencies and genotypes distribution

From a total of 90 subjects, the distribution of CYP2C19 *2/*2, *1/*2, *1/*1, heterozygous *17 (*2/*17 and *1/*17) and *17/*17 were 4.4%, 16.7%, 38.9%, 36.7% and 3.3%, respectively. The CYP2C19*3 allele was not detected. The two single-nucleotide polymorphisms characterizing the CYP2C19*17 allele (806C_T and 3402C _ T) were found in complete linkage disequilibrium in our study population (Table 2).

**Table 2:** CYP2C19, CYP2D6, CYP3A5, AND SULT1A1 genotype frequencies

Genotype	Predicted phenotypes	(n)	% of total	Age (median)	Genotypes	(n)	Genotype frequency
CYP2C19^1^	*2/*2	(4)	4.4	63	*2/*2	(4)	0.03
	*1/*2	(15)	16.7	66	*1/*2	(15)	0.21
	*1/*1	(35)	38.9	62	*1/*1	(35)	0.35
	*1/*17 and *2/*17	(33)	36.7	62	*1/*17	(24)	0.27
					*2/*17	(9)	0.08
	*17/*17	(3)	3.3	65	*17/*17	(3)	0.05
							
CYP2D6^2^	PM	(6)	6.7	65	*4/*4	(4)	0.04
					*4/*5	(2)	0.01
	IM	(32)	35.5	55	*1/*3	(1)	0.01
					*1/*4	(27)	0.29
					*1/*5	(3)	0.04
					*1/*6	(1)	0.01
	Wt/Wt	(51)	56.7	60	*1/*1	(51)	0.48
	UM	(1)	1.1	50	*1/*2 ×2	(1)	
							
CYP3A5	Vt/Vt	(76)	84.4	60	*3/*3	(76)	0.85
	Wt/Vt	(14)	15.6	73	*1/*3	(14)	0.50
	Wt/Wt	(0)			*1/*1	(0)	
							
SULT1A1	Vt/Vt	(14)	15.6	61	*2/*2	(14)	0.11
	Wt/Vt	(32)	35.5	59	*1/*2	(32)	0.44
	Wt/Wt	(44)	48.9	59	*1/*1	(44)	0.45
							
SULT1A1 copy numbers	1	(3)	3.3	59			
	2	(59)	65.6	60			
	3	(21)	23.3	67			
	4	(5)	5.6	72			
	5	(2)	2.2	69			

^1^*2/*2, subjects homozygous for the variant allele CYP2C19*2; *1/*2, subjects heterozygous for the variant allele CYP2C19*2; intermediate metabolizer, subjects homozygous for the wild type allele CYP2C19*1 and with CYP2C19*2/*17; *1/*17 subjects heterozygous for the CYP2C19*17 variant allele; *17/*17 Vt*17 subjects homozygous for the CYP2C19*17 variant allele

^2^PM, poor metabolizer CYP2D6; IM, intermediate metabolizer wild type/variant type CYP2D6; Wt/Wt, wild type/wild type CYP2D6; UM, ultra rapid metabolizer CYP2D6.

The CYP2D6 PM-, IM, Wt/Wt, and UM-genotypes distribution of the patients were 6.7%, 35.5%, 56.7%, and 1.1% respectively (Table 2). 84.4% of the patients carried CYP3A5*3/*3 and 15.6% were CYP3A5*1/*3. The distribution of SULT1A1*1/*1, SULT1A1*1/*2, and SULT1A1*2/*2 were 48.9%, 35.5%, and 15.6% respectively. Three of 90 samples (3.3%) had one copy of the SULT1A1 gene, while 28 patients (31.1%) had three or more copies, with the remaining subjects (65.6%) having two copies (Table 2). Genotypes frequencies of the population met Hardy-Weinberg equilibrium.

### Serum Levels of Tamoxifen, Estrogens, FSH, and SHBG

The serum levels of tamoxifen were positively associated with the serum levels of E2 and E1 (R = 0.295, and 0.297, p = 0.025 and p = 0.020, respectively, Table 3). A positive association was also observed between tamoxifen and E1S (R = 0.254, p = 0.08, Table 3), although it did not reach a level of statistical significance. As shown in Table 3, positive associations were also observed between the serum levels of NDDtam with E2 and E1 (R = 0.315 and R = 0.330, p = 0.01 and p = 0.005, respectively), and tamNox with E1 and E1S (R = 0.292 and R = 0.284, p = 0.025 and p = 0.035, respectively). Furthermore, the serum concentration of 4OHNDtam was associated with the serum level of FSH (R = 0.269, p = 0.05). To minimize a possible effect of CYP2D6 allele variation and to keep a homogenous group, this association was also examined among patients homozygous for the CYP2D6 Wt allele (R = 0.386, p = 0.025). We also observed a negative association between the serum level of E1S (Table 3) and SHBG (R = - 0.445, p < 0.01).

**Table 3:** Associations between the serum concentrations of tamoxifen, estrogens, FSH^1^, and SHBG

Variables	Tamoxifen	4OHtam	4OHNDtam	NDtam	NDDtam	TamNox	SHBG
All (n = 90)	r	r	r	r	r	r	r
E2	0.295*	0.131	0.211	0.148	0.315**	0.232	-0.048
E1	0.297*	0.133	0.152	0.226	0.330**	0.292*	-0.002
E1S	0.254	0.119	0.118	0.247	0.194	0.284*	-0.445**
FSH	0.118	0.065	0.269*	0.134	0.031	0.178	0.095
SHBG	-0.027	0.034	0.088	0.000	0.048	0.103	
							
Wt/Wt EM CYP2D6 (n = 51)							
FSH	0.151	0.133	0.386*	0.148	0.133	0.259	0.224

^1^FSH, Follicle Stimulating Hormone; SHBG, sex hormone binding globulin; 4OHtam, 4-hydroxytamoxifen; 4OHNDtam, 4-hydroxy-N-demethyltamoxifen; NDtam, N-demethyltamoxifen; NDDtam, N-dedimethyltamoxifen; tamNox, tamoxifen-N-oxide; r, coefficient; E2, estradiol; E1, estrone; E1S, estrone sulfate; Wt/Wt EM, wild type/wild type extensive metabolizer CYP2D6.

r and p values are derived from two-tailed Spearman correlation rank test. The p-values are given after Bonferroni correction for multiple (n = 5) comparisons within this data set, *p < 0.05, and **p < 0.01.

### CYP2C19 Genotype

**Figure 2:**
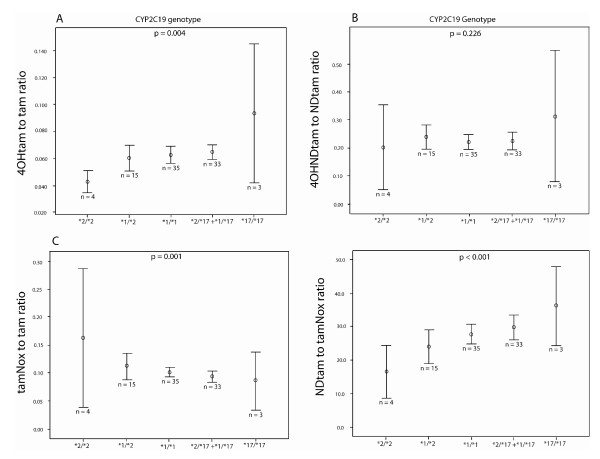
**Relationships between the CYP2C19 genotype and metabolic ratios of 4OHtam/tam, 4OHNDtam/NDtam, tamNox/tam, and NDtam/tamNox**. Error bars represent 95% confidence interval. *2/*2, encodes defective CYP2C19 activity; Vt*17, subjects heterozygous for the variant type allele CYP2C19*17 (*1/*17 and *2/*17); *17/*17, encodes ultra rapid CYP2C19 activity.

The CYP2C19 genotype predicted enzymatic activity was negatively associated with the serum levels of NDDtam and tamNox (p = 0.022 and p = 0.002, respectively) as shown in Table 4. Studying the metabolic ratios of tamoxifen and its metabolites, 4OHtam to tamoxifen (4OHtam/tam) increased with increasing CYP2C19 predicted activity (p = 0.004, figure 2A) whereas no difference was observed between CYP2C19 genotypes and the metabolic ratio of 4OHNDtam to NDtam (p = 0.226, figure 2B). As for the tamNox to tamoxifen ratio was inversely related with CYP2C19 predicted activity (p = 0.001, figure 2C). In contrast the NDtam to tamNox ratio it increased with increasing CYP2C19 predicted activity (p < 0.001, figure 2D).

**Table 4:** Relations between CYP2C19, CYP2D6, CYP3A5 and SULT1A1 genotypes and tamoxifen and its metabolites in postmenopausal women (n = 90)

			Median concentration [q1 - q3] ^1^					
	
Genotype (n)			tamoxifen	4OHtam	4OHNDtam	NDtam	NDDtam	tamNox
CYP2C19^2^	*2/*2	(4)	107 [79 - 137]	5.1 [3.3 - 5.2]	45.7 [41.3 - 54.7]	263 [156 - 404]	47.0 [39.0 - 67.4]	12.8 [10.3 - 31.8]
	*1/*2	(15)	107 [79 - 140]	5.8 [5.1 - 8.9]	61.2 [43.2 - 83.9]	233 [194 - 322]	44.8 [40.2 - 71.4]	9.7 [8.5 - 16.5]
	*1/*1	(35)	92 [79 - 118]	5.5 [4.6 - 6.8]	49.6 [44.7 - 61.6]	231 [190 - 357]	38.1 [26.7 - 46.4]	9.5 [7.4 - 12.7]
	Vt*17	(33)	95 [76 - 126]	6.1 [5.0 - 7.4]	53.3 [41.4 - 67.4]	229 [196 - 319]	37.8 [25.6 - 55.1]	9.1 [6.3 - 10.8]
	*17/*17	(3)	154 [31 - 157]	11.3 [3.0 - 17.2]	94.3 [34.0 - 184.8]	341 [142 - 438]	36.9 [15.7 - 79.0]	11.1 [3.5 - 11.7]
P^3^			0.371	0.240	0.652	0.600	0.022*	0.002**
								
CYP2D6^4^	PM	(6)	85 [58 - 125]	4.0 [3.1 - 5.5]	37.4 [31.8 - 48.2]	296 [179 - 444]	47.0 [22.8 - 55.9]	9.2 [6.1 - 21.0]
	IM	(32)	87 [78 - 141]	5.9 [4.6 - 6.9]	50.5 [38.6 - 62.8]	243 [217 - 358]	38.2 [24.9 - 44.7]	9.4 [7.1 - 11.0]
	Wt/Wt	(51)	99 [80 - 124]	6.1 [5.0 - 8.1]	55.5 [45.7 - 69.7]	223 [117 - 299]	40.4 [33.0 - 55.6]	9.8 [7.4 - 13.4]
	UM	(1)	107	5.8	46.3	185	40.3	9.0
P			0.358	0.019*	0.009**	0.044*	0.413	0.958
								
CYP3A5	Vt/Vt	(76)	96 [75 - 127]	5.6 [4.7 - 7.0]	49.9 [41.3 - 65.5]	228 [189 - 316]	38.4 [27.0 - 51.4]	9.4 [6.9 - 11.8]
	Wt/Vt	(14)	102 [81 - 162]	6.8 [5.7 - 10.2]	54.6 [49.4 - 80.6]	297 [221 - 396]	45.7 [32.7 - 67.3]	12.0 [9.7 - 14.5]
P			0.203	0.074	0.154	0.075	0.052	0.044*
								
SULT1A1	Vt/Vt	(14)	91 [68 - 138]	5.5 [4.1 - 7.5]	48.6 [40.4 - 60.7]	228 [185 - 353]	37.1 [23.4 - 49.8]	9.3 [6.9 - 11.8]
	Wt/Vt	(32)	103 [90 - 141]	6.0 [5.1 - 7.3]	58.5 [46.2 - 75.3]	244 [201 - 361]	44.8 [37.9 - 57.6]	10.7 [8.7 - 15.2]
	Wt/Wt	(44)	87 [80 - 105]	5.4 [4.6 - 8.9]	51.3 [40.8 - 73.5]	219 [183 - 294]	36.9 [25.7 - 49.4]	9.0 [6.8 - 10.1]
P			0.649	0.512	0.286	0.755	0.197	0.712
								
SULT1A1copy numbers	1	(3)	84 [80 - 168]	6.7 [6.1 - 8.9]	55.1 [49.0 - 85.0]	278 [217 - 364]	37.9 [24.2 - 60.4]	9.7 [6.9 - 10.6]
	2	(59)	92 [68 - 123]	5.6 [4.6 - 6.8]	50.5 [41.2 - 64.4]	227 [188 - 299]	38.4 [27.7 - 47.9]	9.4 [6.6 - 11.7]
	3	(21)	107 [87 - 162]	6.9 [5.2 - 9.8]	52.7 [46.6 - 80.2]	271 [198 - 395]	46.5 [33.0 - 59.2]	9.8 [8.3 - 13.8]
	4	(5)	103 [82 - 139]	5.1 [4.2 - 7.5]	58.6 [39.1 - 71.7]	223 [205 - 346]	38.1 [28.4 - 62.3]	12.7 [8.4 - 14.5]
	5	(2)	86 [51 - 122]	6.2 [4.1 - 8.4]	49.2 [32.4 - 66.1]	260 [150 - 371]	45.1 [23.1 - 67.2]	11.7 [5.6 - 17.7]
P^4^			0.352	0.863	0.719	0.475	0.370	0.280

^1^Median concentration [quartile 1 - quartile 3] values of tamoxifen and metabolites are given as ng/ml.

^2^*2/*2, encodes defective CYP2C19 activity; Vt*17, subjects heterozygous for the variant type allele CYP2C19*17 (*1/*17 and *2/*17); *17/*17, encodes ultra rapid CYP2C19 activity.

^3^P values were calculated by multivariate logistic regression model, *p < 0.05. Adjusted for CYP2C19, CYP2D6, CYP3A5, SULT1A1, SULT1A1 copy number, FSH and SHBG.

^4^PM, poor metabolizer; IM, wild type/variant type; Wt/Wt, wild type/wild type; UM, ultra rapid metabolizer.

Of the 15 individuals with predicted low CYP2C19*1/*2 enzymatic activity, 1 had CYP2D6 PM status, 5 IM status, 8 Wt/Wt status, and 1 UM status. In this CYP2C19*1/*2 subgroup, we observed no significant difference for the 4OHtam/tam ratio between the groups based on CYP2D6 status (p = 0.932, data not shown). Furthermore, 35 individuals had CYP2C19*1/*1, of these 2 had PM status, 11 IM status, and 22 Wt/Wt status. There was a positive association between the ratio of 4OHtam/tam and CYP2D6 gene dose effect (p = 0.036, data not shown). Moreover, we observed 33 individuals heterozygous for the CYP2C19*17 allele, of these 1 with PM status, 16 CYP2D6 IM status, and 16 CYP2D6 Wt/Wt status. There was also no significant difference for 4OHtam/tam observed between these groups (p = 0.403). Thus, our results suggest that CYP2D6 status does affect the ratio of 4OHtam/tam only in subjects with normal CYP2C19 activity, but not in the subjects carrying CYPC19 *17 allele.

Studying the influence of CYP2C19 genotypes on estrogen metabolism we observed a negative association between increased CYP2C19 predicted enzymatic activity and E1 (Table 5). Lower serum levels of E1 was observed in patients either heterozygous or homozygous carriers of the CYP2C19*17 allele when compared with patients either heterozygous or homozygous for the CYP2C19*2 allele (p = 0.019), as shown in Table 5. No associations were observed between FSH, SHBG and CYP2C19 (data not shown).

**Table 5:** Relations between CYP2C19, CYP2D6, CYP3A5 and SULT1A1 genotypes and estrogens in postmenopausal women (n = 90)

			Median concentration [q1 - q3] ^1^		
			
Genotype (n)			E1	E2	E1S
CYP2C19^2^	*2/*2	(4)	122 [88 - 303]	9.6 [6.7 - 43.5]	857 [309 - 1400]
	*1/*2	(15)	106 [72 - 182]	13.0 [6.6 - 32.0]	391 [234 - 955]
	*1/*1	(35)	102 [70 - 124]	10.9 [5.7 - 16.0]	655 [345 - 928]
	Vt*17	(33)	86 [66 - 104]	8.4 [6.0 - 12.1]	545 [259 - 801]
	*17/*17	(3)	75 [54 - 122]	9.3 [9.3 - 17.4]	428 [158 - 654]
P^3^			0.019*	0.139	0.227
					
CYP2D6^4^	PM	(6)	111 [96 - 154]	9.4 [6.6 - 24.4]	491 [318 - 878]
	IM	(32)	91 [66 - 111]	7.7 [5.2 - 14.1]	595 [255 - 931]
	Wt/Wt	(51)	101 [67 - 128]	10.9 [6.1 - 17.4]	588 [345 - 888]
	UM	(1)	72	6.6	391
P			0.559	0.938	0.804
					
CYP3A5	Vt/Vt	(76)	99 [71 - 123]	10.5 [6.6 - 16.4]	569 [305 - 865]
	Wt/Vt	(14)	97 [65 - 124]	8.0 [4.3 - 10.5]	631 [294 - 1127]
P			0.520	0.248	0.221
					
SULT1A1	Vt/Vt	(14)	80.6 [66.4- 103.5]	6.7 [5.2- 9.4]	590.4 [316.5- 851.1]
	Wt/Vt	(32)	99.1 [68.6-121.7]	13.7 [5.3-18.9]	537.0 [318.0- 768.7]
	Wt/Wt	(44)	102.1 [70.9-142.1]	9.4 [1.4-54.7]	608.5 [135.6-2922.9]
P			0.280	0.575	0.734
					
SULT1A1copy numbers	1	(3)	59.7 [58.7-101.2]	3.8 [2.6- 6.5]	252.9 [235.5- 939.0]
	2	(59)	89.8 [67.2-110.2]	7.7 [5.5-15.2]	498.4 [271.6- 758.9]
	3	(21)	105.7 [90.7-128.6]	11.8 [9.0-14.3]	859.5 [561.3- 955.2]
	4	(5)	101.8 [98.0- 103.0]	15.3 [12.6-19.1]	363.6 [227.5- 662.0]
	5	(2)	157.6 [155.3-160.0]	13.6 [9.4-17.9]	2086.3 [1780.7-2391.9]
P			0.024*	0.010*	0.005**

^1^Median concentration [quartile 1 - quartile 3] values of tamoxifen and metabolites are given as ng/ml.

^2^*2/*2, encodes defective CYP2C19 activity; Vt*17, subjects heterozygous for the variant type allele CYP2C19*17 (*1/*17 and *2/*17); *17/*17, encodes ultra rapid CYP2C19 activity.

^3^P values were calculated by multivariate logistic regression model, *p < 0.05. Adjusted for CYP2C19, CYP2D6, CYP3A5, SULT1A1, SULT1A1 copy number, FSH and SHBG.

^4^PM, poor metabolizer; IM, wild type/variant type; Wt/Wt, wild type/wild type; UM, ultra rapid metabolizer.

### CYP2D6 Genotype

We have shown earlier that CYP2D6 genotypes influenced tamoxifen kinetics in a dose dependent manner [7]. In this sub-group of postmenopausal women, we observed positive relations between the serum levels of 4OHtam and 4OHNDtam and CYP2D6 gene dose effect (p = 0.019 and p = 0.009, respectively, Table 4). In contrast, NDtam was inversely associated with CYP2D6 predicted increasing activity (p = 0.044). We observed no relationship between the serum levels of estrogens and CYP2D6 genotypes (Table 5), and no associations were observed between FSH, SHBG and CYP2D6 (data not shown).

### CYP3A5 Genotype

We also examined the association between the CYP3A5 genotypes and serum levels of tamoxifen and its metabolites. The median serum concentration of tamNox was lower for patients homozygous for the CYP3A5*3 allele than heterozygous CYP3A5*1/*3, (p = 0.04, Table 4). The same trend was observed in the serum levels of 4OHtam, NDtam and NDDtam, although these did not reach significant values (p = 0.074, p = 0.075 and p = 0.052, respectively). There was no association observed between the serum concentrations of E2, E1, E1S, and the CYP3A5 genotypes (Table 5). The same were observed between FSH, SHBG and CYP3A5 (data not shown).

### SULT1A1 Genotype

No association between the serum levels of tamoxifen and its metabolites and the SULT1A1 genotypes was demonstrated in this subgroup of postmenopausal women. The serum concentrations of E2, E1, and E1S were not associated with SULT1A1 allele variation (Table 5). However SULT1A1 copy number was positively associated with the serum levels of E2, E1, and E1S (p = 0.024, p = 0.010, and p = 0.005; respectively). The SULT1A1 genotypes did not influence FSH and SHBG (data not shown).

## Discussion

In this study we used two highly sensitive assays for measuring the serum levels of tamoxifen and its metabolites and estrogens [28,29]. As hypothesized, we observed positive associations between the serum levels of estrogens (E1 and E2) and tamoxifen, as well as two of its' non-hydroxylated metabolites (NDDtam and tamNox). Interestingly, the concentrations of the more potent hydroxylated tamoxifen metabolites (4OHtam and 4OHNDtam) were not correlated to estrogen levels.

Metabolites of tamoxifen that are not hydroxylated may also contribute to the effects or adverse effects of tamoxifen. NDtam, NDDtam and tamNox have estrogen receptor affinity that is the same as for tamoxifen itself but which is 100-fold lower than 4OHtam and 4OHNDtam [2,31,32]. Notably, high levels of NDtam and NDDtam have been reported in patients experiencing tamoxifen-related side effects [33,34]. In animals NDDtam appears to decrease the rate of tamoxifen metabolism [35] whereas tamNox may represent a pool for its' reconversion back to tamoxifen [36]. The roles of NDtam, NDDtam, and tamNox in the clinical situation remain to be explored more in detail.

Dowsett *et al *suggests that tamoxifen effectively saturates ER during tamoxifen of postmenopausal women [31]. This may suggest that estrogen levels during tamoxifen therapy may have limited importance. However, long term exposure to tamoxifen has been shown to induce a state of adaptive hypersensitivity in breast tumors to E2 [22]. Thus, upon development of tamoxifen resistance, low levels of estrogen may stimulate tumor growth. Moreover, clinical studies have shown that the expression of 17β-hydroxysteroid dehydrogenase 1 (17HSD1), which converts E1 to the active estrogen E2, and 17HSD2, which converts E2 to E1, are predictive factors for treatment response to tamoxifen in both premenopausal and postmenopausal patients [37,38]. These suggest that estrogen levels may be of importance during long term tamoxifen therapy.

Tamoxifen and estrogens are both partly metabolized by the enzymes CYP2C19, 2D6, 3A5, and SULT1A1 [27]. Therefore, we screened for associations between these genotypes and estrogen serum levels and observed that the predicted CYP2C19 activity was related to the level of E1. This should be expected as only E1 and not E2 or E1S is converted by CYP2C19 (figure 1B). Patients carrying the CYP2C19*17 allele predicting high enzyme activity had lower serum levels of E1 compared with those carrying CYP2C19 defective alleles. This finding is in agreement with a recent report suggesting that an increased catabolism of estrogens by CYP2C19 may lead to decreased estrogen levels and therefore reduced breast cancer risk [39], and the observation that CYP2C19*17 identifies patients likely to benefit from tamoxifen treatment [19].

The 4OHtam to tamoxifen ratio was positively associated with increasing CYP2C19 predicted enzymatic activity, whereas no such correlation was observed for the ratio of 4OHNDtam to NDtam. This is consistent with previous reports showing that CYP2C19 hydroxylates tamoxifen, but not NDtam [40]. Of note is the finding that the metabolic ratio NDtam/tamNox was highly significantly related to the predicted activity of CYP2C19. This is in line with the suggestion of Jordan *et al *that tamNox represents an intermediate metabolic step between tamoxifen and NDtam [41].

The predicted CYP2D6 and CYP3A5 activities did not influence estrogen levels, whereas SULT1A1 gene copy number was positively related to the levels of E2, E1, and E1S, but not to those of tamoxifen or its metabolites. A novel finding in the present study was the observation of lower serum levels of tamNox in patients homozygous for the low activity allele CYP3A5*3 compared to patients heterozygous for this allele. Although not significant there was also a trend for lower levels of 4OHtam, NDtam and NDDtam. This was expected since CYP3A5 hydroxylates and demethylates tamoxifen (figure 1A). Wegman *et al *observed, in contrast to Goetz *et al*, that patients homozygous for CYP3A5*3 have improved recurrence free-survival [18,25]. The clinical importance of CYP3A5 in this matter remains unanswered.

The observed associations between the kinetics of tamoxifen and the levels of estrogens may be due to competition between tamoxifen or its metabolites and estrogens to be processed by identical enzymes such as CYP2C19. An alternative explanation is that tamoxifen and its metabolites inhibit enzymes that are involved in the degradation of estrogens. This is line with the finding of Meltzer *et al *who demonstrated that tamoxifen and NDtam may act both competitively and non-competitively on microsomal mixed function oxidases [42]. Another explanation is that the associations may also be a consequence of the observed increase of serum levels of dehydroepiandrosterone (DHEA) during tamoxifen treatment therapy [43,44]. DHEA can be converted to androstendione which is a precursor of estrogens. Both tamoxifen and estrogens are also excreted in bile as conjugates. After deconjugation by intestinal microflora they are partly reabsorbed resulting in pronounced enterohepatic cycling [45,46]. Accordingly, the deconjugation capacity of the microflora in the intestines may be a common determinant for their re-absorption and bioavailability [6,47].

We have observed estrogen agonistic effects of tamoxifen in the liver and in the pituitary gland of postmenopausal patients [48]. The serum level of SHBG was more than doubled and the level of FSH was almost halved. To be certain that tamoxifen levels were in steady-state in the present study all patients included were treated with tamoxifen for at least 80 days. After this treatment period we believe that also estrogen levels were at steady state levels. Although tamoxifen exerts an estrogen agonistic effect on the pituitary of postmenopausal women, we do not know the separate effects of each of its metabolites on the pituitary. Therefore, it is interesting that of the five tamoxifen metabolites examined, only the serum concentrations of 4OHNDtam was positively associated with the serum levels of FSH in this situation of steady-state tamoxifen kinetics. This suggests that 4OHNDtam has an anti-estrogenic effect in the pituitary gland.

Hot flashes have been suggested as a predictor of tamoxifen efficacy [26]. Of note, FSH serum concentrations correspond better with the frequency of hot flashes than the levels of E2 [23]. Interestingly, some results from clinical studies suggest that patients carrying functional CYP2D6 alleles have a higher incidence of hot flashes, higher levels of 4OHNDtam and better outcome during tamoxifen treatment [25,26]. Accordingly, relations between the frequency of hot flashes, serum concentrations of 4OHNDtam and outcome during tamoxifen therapy may exist.

Limitations of the present study are that the correlations observed are weak and we have not had admittance to patient journals and do not know of any additional drug use. However, we and others have observed that breast cancer patients often use several additional drugs that may interact with tamoxifen [49-52]. Accordingly, the weak correlations observed are not surprising as drugs, enzymes and other factors in addition to those analyzed in the present study may interact with the metabolism of tamoxifen as well as that of estrogens.

## Conclusions

We have shown an association between the non-hydroxylated metabolites of tamoxifen and estrogen serum levels. The influence of CYP2C19 predicted activity on tamoxifen and estrogen kinetics may partly explain this observation. Thus estrogens as well as tamoxifen metabolites may influence the observed tamoxifen treatment benefit of patients carrying CYP2C19 *17 variant. In addition, the more potent hydroxylated tamoxifen metabolite 4OHNDtam (endoxifen) was the only tamoxifen metabolite positively associated with FSH levels suggesting anti-estrogenic effect on the pituitary. This may explain the observed positive association between a better prognosis and FSH levels during tamoxifen therapy. Our results may be of importance for the effects, side effects, timing and duration of tamoxifen treatment. Further prospective studies to examine the effect of tamoxifen and estrogen kinetics on treatment outcome are therefore warranted.

## Competing interests

The authors declare that they have no competing interests.

## Authors' contributions

JG^1^ conceived the study, participated in the design of the study, performed the quantifications of tamoxifen and metabolites, CYP2C19 and SULT1A1 genotyping, statistical analysis of the data and drafted the manuscript. JG and DE participated in performing estrogen measurements and drafted the manuscript. SL and JEV conceived the study, participated in the design of the study, collected the materials and drafted the manuscript. VMS conceived the study, participated in the design of the study, performed the CYP2D6 genotyping, statistical analysis of the data and drafted the manuscript. GM and EAL conceived the study, participated in the design of the study, statistical analysis of the data and drafted the manuscript. All authors read and approved the final manuscript.

## Pre-publication history

The pre-publication history for this paper can be accessed here:

http://www.biomedcentral.com/1471-2407/10/313/prepub
